# Development of a clinical nomogram for predicting sarcopenia in patients with chronic obstructive pulmonary disease based on NHANES data

**DOI:** 10.3389/fmed.2025.1612403

**Published:** 2025-07-30

**Authors:** Xingfu Fan, Jin Zhao, Yang Luo, Xiaofang Li, Wenqin Tan, Shiping Liu

**Affiliations:** Department of General Medicine, Affiliated Hospital of North Sichuan Medical College, Nanchong, Sichuan, China

**Keywords:** COPD, sarcopenia, nomogram, NHANES, risk factors

## Abstract

**Background:**

The prevalence of sarcopenia in COPD patients is high, and the mutual influence between COPD and sarcopenia creates a vicious cycle. The goal of this research is to create a nomogram model that can forecast when sarcopenia will strike people with COPD.

**Methods:**

2011 to 2018 data were retrieved from four NHANES database cycles. The 7:3 proportion was applied to split the dataset randomly to separate validation and training datasets. Multivariate logistical regression and LASSO regression were applied to design nomogram design and to select predictors. In addition, multicollinearity existence among final predictor variables remaining in model were tested, among other variables. Calibration curve, decision curve analysis (DCA), and area under receiver operating characteristic curve (AUC) were applied in testing performance in prediction model.

**Results:**

The nomogram was constructed based on four predictive factors: gender, height, BMI, and WWI. The AUC for the training set was 0.94 (95% CI 0.91–0.97), and the AUC for the validation set was 0.91 (95% CI 0.83–0.98), indicating excellent predictive performance. Furthermore, the clinical applicability of the model has been thoroughly validated.

**Conclusion:**

We established a nomogram model to provide an easy and convenient way for early screening of sarcopenia in COPD patients, and to allow for effective guidance to perform early intervention and manage patient prognosis in an optimal way.

## 1 Introduction

Chronic Obstructive Pulmonary Disease (COPD) is an inflammatory condition, with gradual progression that involves the airway, alveoli, and microvasculature (1). The most recent Global Burden of Disease report has shown that COPD is the fourth cause of death globally, with a global mortality of 45.2/100,000 population (2). There are projected increases of 23% in the number of cases of COPD for those 25 years of age and older to 2050 amounting to 600 million cases globally. Low- and middle-income countries are estimated to have a prevalence over two times greater than that of high-income countries (3). Beyond localized lung inflammation, COPD can also induce systemic changes through systemic inflammation (4). These changes include reduced physical activity levels, as well as a loss of muscle mass and strength, all of which are closely associated with the development of sarcopenia (5, 6). Sarcopenia is significantly linked to increased risks of falls, hospitalization, and mortality (7, 8). The main characteristic of this muscle tissue disease is the progressive decrease of mass as well as strength (9). Global surveys reveal that the prevalence of sarcopenia ranges from 10 to 27%. Regionally, Oceania exhibits the highest prevalence at approximately 40%, followed by South America at 35% (10).

Over the last years, the pathophysiology of the coexistence between COPD and sarcopenia has received more and more attention, presenting among the most common COPD comorbidities. COPD patients contribute to the pathogenesis of sarcopenia by different mechanisms such as inflammation (4), oxidative stress (11), and disuse of skeletal muscles (12). Research has demonstrated that the prevalence of sarcopenia in stable COPD patients is 14.5%, and the prevalence increases gradually with age and disease severity (5). Another 124 patients with COPD, who were recruited in a cross-sectional study, reported prevalence of pre-sarcopenia of 46.3% and of sarcopenia of 12.4% (13). Moreover, in COPD patients sarcopenia strongly increases the chance of a bad prognosis. A prospective study conducted by the National Institute of Respiratory Diseases in Mexico among 240 COPD patients followed for 6.6 years, identified sarcopenia, low muscle strength and low grip strength as independent risk factors for poor prognosis in COPD patients (14). In addition, one study with data from the National Health and Nutrition Examination Survey (NHANES) reported an evidence that sarcopenia was significantly associated with greater all-cause mortality in US patients with COPD (15). The interaction of COPD and Sarcopenia induces a vicious cycle which aggravates the disease progress and increases both physical and mental burden to the patients, leading to a critically poor health status.

Although the prevalence of sarcopenia in patients with COPD is high, studies have shown that early identification and the use of nutritional supplements can significantly improve weight, muscle mass, and physical functions in COPD patients, such as the 6-min walk test, physical activity levels, and the 5-time sit-to-stand test (16). Thus, early identification, diagnosis, and treatment of sarcopenia in individuals with COPD may improve their prognosis and quality of life by recovering their functional ability and easing physical and mental health problems. However, according to the National Institute of Health (NIH) Foundation’s statement on the diagnosis of sarcopenia, current diagnostic methods require the measurement of appendicular lean body weight divided by body mass index (BMI). This diagnostic approach is not only cumbersome but also difficult to implement in some primary healthcare settings due to limited equipment. To date, nomogram models for the diagnosis of sarcopenia in COPD patients have not been widely reported. The advantage of nomograms lies in their ability to transform complex regression models into intuitive visual graphs, which help clinicians assess patient risk more conveniently and directly. As a result, nomograms have been widely applied in clinical settings for the assessment of disease risk and prognosis.

Building on this background, the purpose of this research is to create a nomogram model that can forecast a patient’s chance of developing sarcopenia if they have COPD. This model is designed to assist clinicians in early, accurate, and efficient identification of COPD patients with concurrent sarcopenia. By enabling timely detection, clinicians will be able to implement appropriate interventions, ultimately improving patient prognosis. This study holds significant clinical value, as it provides scientific evidence to guide the management of COPD patients and optimize treatment strategies.

## 2 Materials and methods

### 2.1 Study participants

**FIGURE 1:**
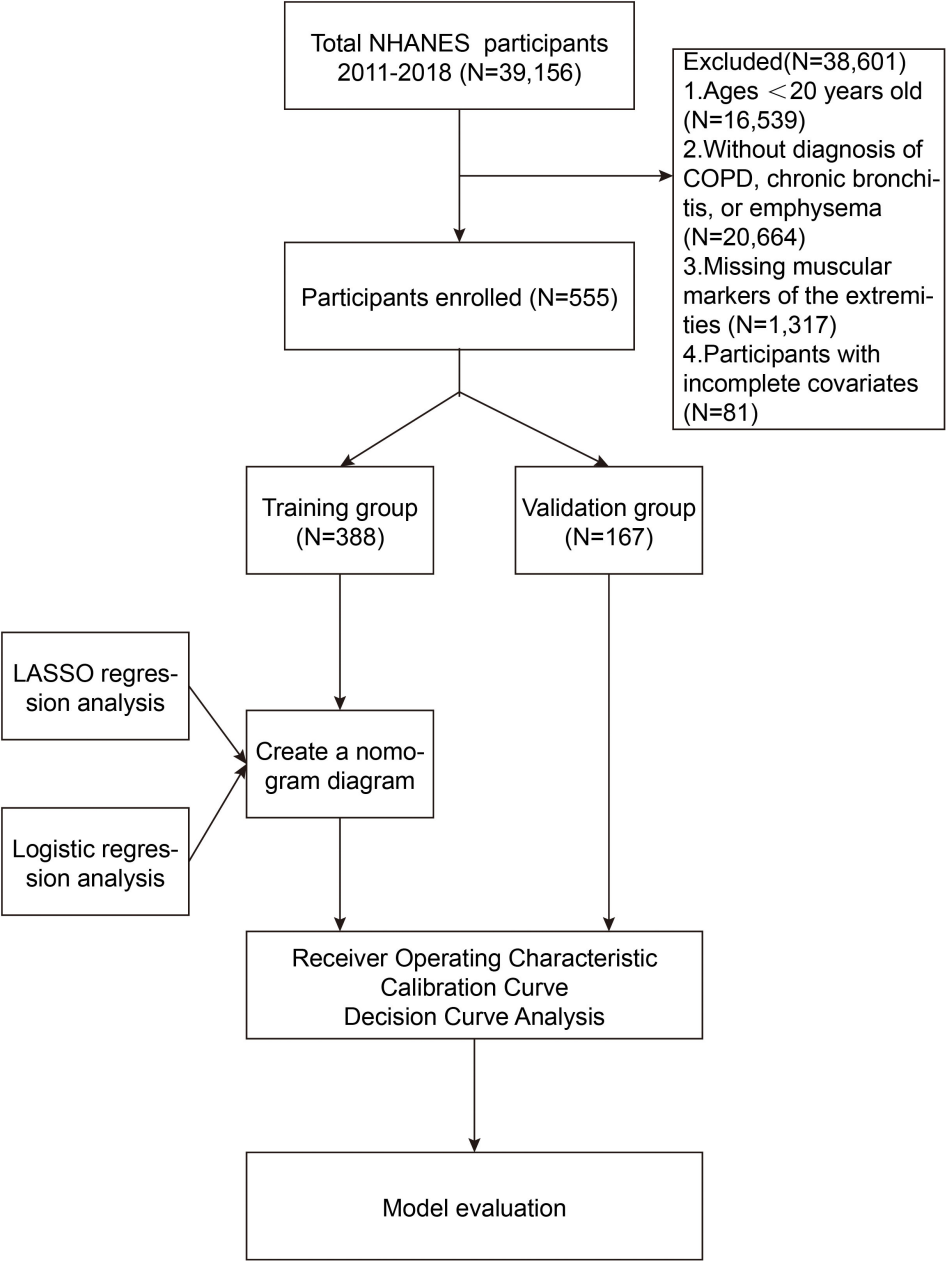
The flow chart of study population screening and statistical analysis.

The NHANES is an ongoing program designed to collect comprehensive data on the health and nutritional status of the U.S. population. Through interviews, physical examinations, and laboratory tests, NHANES provides valuable data on chronic diseases, risk factors, dietary habits, and other health-related aspects. This study is retrospective in nature, utilizing publicly available data, which eliminates the need for Institutional Review Board (IRB) approval. The analysis in this study used NHANES data from four cycles between 2011 and 2018, including a total of 39,156 participants. The following were the removal criteria used in this study: (1) Individuals with a weight exceeding 136 kg or a height greater than 192 cm, due to limitations of dual-energy X-ray absorptiometry (DXA) scanning; (2) pregnant ladies and people with contrast agent allergies; (3) those who had radiation therapy or contrast chemicals within the previous 7 days; (4) Participants under the age of 20 (*N* = 16,539); (5) Participants who had not been diagnosed with COPD, chronic bronchitis, or emphysema (*N* = 20,664); (6) Participants lacking data on appendicular skeletal muscle mass (*N* = 1,317); (7) Participants missing data on relevant covariates (*N* = 81). Further details are provided in Figure 1.

### 2.2 Diagnosis criteria for COPD

Due to the lack of direct COPD-related questionnaires during the 2011–2012 cycle, this study adopted diagnostic criteria based on previous research (17, 18). The criteria were as follows: (1) During the 2013–2018 survey cycles, participants answered “Has a doctor ever diagnosed you with COPD?”, “Has a doctor informed you that you have chronic bronchitis?”, or “Have you been told by a doctor that you have emphysema?” (answered “yes”); (2) During the 2011–2012 cycle, participants were informed of having emphysema or chronic bronchitis, or had a forced expiratory volume in 1 s (FEV1) to forced vital capacity (FVC) ratio of less than 0.7 after using a bronchodilator.

### 2.3 Diagnosis criteria for sarcopenia

Appendicular skeletal muscle mass, as a critical biomarker for assessing lean body mass in the limbs, is widely recognized in clinical practice. This study utilized the Skeletal Muscle Index (SMI), as recommended by the NIH Sarcopenia Project. The SMI is calculated by dividing appendicular skeletal muscle mass (ASM) by BMI (19). According to the NIH evidence-based diagnostic criteria, the threshold for diagnosing low muscle mass was defined as SMI < 0.789 for males and <0.512 for females. Individuals meeting these criteria were included in the sarcopenia study cohort (20).

### 2.4 Measurement and collection of covariates

We systematically collected demographic and lifestyle data, including actual age, gender, race, marital status, educational attainment, and the ratio of poverty to income (PIR, with low income defined as ≤1, middle income as >1 and ≤3, and high income as >3), smoking status (never smoked [<100 cigarettes], former smoker [>100 cigarettes and quit], current smoker [>100 cigarettes and smoking during the survey period]), alcohol consumption (defined as drinking at least 12 alcoholic beverages in the past year, with others considered non-drinkers), height, weight, BMI, arm circumference, waist circumference (WC), physical activity (The Global Physical Activity Questionnaire [GPAQ] was used for assessment (21). In accordance with World Health Organization (WHO) guidelines, physical activity was measured in metabolic equivalent minutes (MET-min), where ≥600 MET-min/week was considered acceptable and <600 MET-min/week was considered insufficient.) and the weight-adjusted waist circumference index (WWI, calculated as WC [cm] divided by the square root of weight [kg]).

In the laboratory data module, we collected potential predictive indicators, including 25-OHD3, alanine aminotransferase (ALT), aspartate aminotransferase (AST), alkaline phosphatase (ALP), serum albumin (ALB), blood urea nitrogen, creatine kinase, serum creatinine, total serum protein, total calcium, uric acid, glycated hemoglobin (HbA1c), white blood cell count (WBC), lymphocyte count, monocyte count, neutrophil count, red blood cell count, hemoglobin, platelets, triglycerides, high-density lipoprotein, systemic immune-inflammation index [SII, calculated as SII = peripheral platelet count (×10^3^cells/μL) × neutrophil count (×10^3^cells/μL)/lymphocyte count (×10^3^cells/μL)] according to previous research (22), and nutritional risk index [GNRI, calculated as GNRI = (1.489 × albumin [g/L]) + 41.7 × (actual weight/ideal weight)]. In addition, we also collected the monocyte-to-lymphocyte ratio (MLR), calculated as the monocyte count divided by the lymphocyte count, based on previous research.

Moreover, participants’ medical history information was collected by asking whether they had been diagnosed by a healthcare professional. Cardiovascular disease (CVD) diagnosis was based on one of the following: congestive heart failure, heart disease, angina, coronary artery disease, or stroke; diabetes diagnosis was based on one of the following: (1) HbA1c ≥ 6.5%; (2) fasting blood glucose ≥ 7 mmol/L; (3) A self-reported history of hypertension or current use of antihypertensive medication, as well as an average systolic blood pressure measurement of 130 mmHg or an average diastolic blood pressure measurement of 80 mmHg over three readings, were used to diagnose hypertension.

### 2.5 Statistical methods

R (version 4.3.2) was used to conduct the statistical study. Both continuous and categorical variables were handled in descriptive statistical analysis. The Wilcoxon rank-sum test or *t*-tests were used for intergroup comparisons of continuous variables. Categorical variables were compared using the chi-square test or Fisher’s exact test. The Least Absolute Shrinkage and Selection Operator (LASSO) was applied first as a preliminary step of the predictor selection. Then multivariate logistic regression analysis was conducted to determine the final predictive factors for sarcopenia in COPD patients. Furthermore, possible multicollinearity of the final predictor variables in the model was also examined. Nomogram of the model was constructed by the rms package to visualize the model. To evaluate the predictive ability of the model, we conducted receiver operating characteristic (ROC) curve analysis, in which an area under the curve (AUC) value of ≥0.75 was considered strong discrimination (23). Internal validation of the nomogram prediction model was performed using 1000 bootstrap resampling repetitions, while the predictive accuracy and clinical utility of the model were both assessed by calibration curves and Decision Curve Analysis (DCA). A *p*-value < 0.05 was defined as statistically significant in the present study.

## 3 Results

### 3.1 Participant characteristics

A total of 555 COPD patients were included in this study, with their baseline characteristics presented in Table 1. Using R software, 555 COPD patients were randomly divided into a training group and a validation group in a 7:3 ratio. The training group consisted of 388 patients, while the validation group included 167 patients. Based on statistical analysis, all variables were included in the subsequent development of the clinical nomogram prediction model.

**TABLE 1:** The comparison of patient characteristics between the training group and validation group.

Characteristic	Total (*N* = 555)	Validation group (*N* = 167)	Training group (*N* = 388)	*P-*value
Age (years)	47.00 (35.50, 55.00)	47.00 (37.50, 55.00)	46.00 (35.00, 54.00)	0.170
25-OHD_3_ (nmol/L)	58.80 (39.20, 76.70)	60.73 (35.52, 78.75)	58.10 (40.30, 76.15)	0.940
Alanine aminotransferase (U/L)	20.00 (15.00, 28.00)	20.00 (16.00, 27.50)	19.00 (15.00, 28.00)	0.683
Aspartate aminotransferase (U/L)	21.00 (18.00, 26.00)	22.00 (19.00, 26.50)	21.00 (18.00, 26.00)	0.105
Albumin (g/L)	42.00 (40.00, 44.00)	42.00 (40.00, 44.00)	42.00 (40.00, 44.00)	0.802
Alkaline phosphatase (IU/L)	69.00 (55.00, 86.00)	70.00 (56.50, 87.50)	68.00 (55.00, 85.25)	0.500
Blood urea nitrogen (mg/dL)	12.00 (9.00, 14.00)	12.00 (9.00, 15.00)	12.00 (9.00, 14.00)	0.946
Creatine phosphokinase (IU/L)	103.00 (69.00, 153.50)	104.00 (69.00, 194.50)	103.00 (69.00, 140.00)	0.069
Creatinine, serum (umol/L)	71.60 (61.88, 84.86)	72.49 (63.65, 83.10)	71.60 (61.88, 85.75)	0.856
Total calcium (mmol/L)	2.35 (2.27, 2.40)	2.35 (2.27, 2.40)	2.35 (2.27, 2.40)	0.412
Total albumin (g/L)	71.00 (67.00, 74.00)	71.00 (68.00, 75.00)	70.00 (67.00, 74.00)	0.194
Uric acid (mg/dL)	309.30 (255.80, 362.80)	309.30 (261.70, 362.80)	309.30 (255.80, 368.80)	0.984
Glycohemoglobin (%)	5.50 (5.30, 5.90)	5.60 (5.30, 6.00)	5.50 (5.30, 5.80)	0.136
White blood cell count (1000 cells/uL)	7.50 (6.10, 9.30)	7.50 (6.20, 9.50)	7.50 (6.10, 9.12)	0.857
Lymphocyte number (1000 cells/uL)	2.20 (1.80, 2.70)	2.30 (1.80, 2.70)	2.20 (1.80, 2.70)	0.524
Monocyte number (1000 cells/uL)	0.60 (0.45, 0.70)	0.50 (0.40, 0.70)	0.60 (0.50, 0.70)	0.768
Neutrophils number (1000 cells/uL)	4.40 (3.30, 5.70)	4.20 (3.35, 5.80)	4.45 (3.30, 5.70)	0.942
Red blood cell count (million cells/uL)	4.66 (4.34, 5.01)	4.61 (4.28, 5.04)	4.67 (4.37, 4.97)	0.594
Hemoglobin (g/dL)	14.00 (13.10, 15.10)	13.90 (13.05, 15.00)	14.00 (13.10, 15.12)	0.456
Platelet count (1000 cells/uL)	246.00 (209.50, 285.00)	251.00 (210.50, 291.00)	244.00 (207.75, 281.00)	0.258
Triglyceride (mmol/L)	4.97 (4.32, 5.74)	5.09 (4.37, 5.93)	4.91 (4.27, 5.61)	0.083
Direct HDL-cholesterol (mmol/L)	1.24 (1.01, 1.50)	1.24 (1.04, 1.65)	1.22 (0.98, 1.48)	0.127
Weight (kg)	82.00 (68.60, 97.60)	84.10 (67.85, 100.20)	80.60 (68.97, 97.12)	0.498
Height (cm)	166.20 (160.25, 172.55)	165.50 (159.50, 173.65)	166.40 (160.40, 172.22)	0.784
BMI (kg/m^2^)	29.40 (24.45, 35.65)	30.70 (24.85, 36.60)	29.10 (24.20, 35.40)	0.434
Arm circumference (cm)	33.80 (30.30, 37.60)	34.80 (29.75, 38.00)	33.50 (30.30, 37.32)	0.235
Waist circumference (cm)	101.00 (89.20, 114.80)	102.20 (90.70, 116.10)	100.30 (88.68, 113.35)	0.538
SII	488.84 (342.11, 676.25)	486.94 (346.91, 701.14)	490.50 (333.91, 667.91)	0.685
MLR	0.25 (0.20, 0.31)	0.25 (0.20, 0.30)	0.25 (0.20, 0.32)	0.540
WWI (cm/√kg)	11.13 (10.62, 11.73)	11.18 (10.68, 11.80)	11.11 (10.60, 11.70)	0.517
Gender, *n* (%)				0.400
Male	204 (36.76)	57 (34.13)	147 (37.89)	
Female	351 (63.24)	110 (65.87)	241 (62.11)	
Race (%)				0.923
Mexican American	31 (5.59)	8 (4.79)	23 (5.93)	
Other Hispanic	51 (9.19)	14 (8.38)	37 (9.54)	
Non-Hispanic White	300 (54.05)	89 (53.29)	211 (54.38)	
Non-Hispanic Black	107 (19.28)	35 (20.96)	72 (18.56)	
Other race	66 (11.89)	21 (12.57)	45 (11.60)	
Education *n* (%)				0.321
<High school	124 (22.34)	32 (19.16)	92 (23.71)	
High school	143 (25.77)	49 (29.34)	94 (24.23)	
>High school	288 (51.89)	86 (51.50)	202 (52.06)	
Marriage, *n* (%)				0.078
Married	234 (42.16)	61 (36.53)	173 (44.59)	
Non-married	321 (57.84)	106 (63.47)	215 (55.41)	
Family income, *n* (%)				0.221
Low	179 (32.25)	48 (28.74)	131 (33.76)	
Medium	254 (45.77)	75 (44.91)	179 (46.13)	
High	122 (21.98)	44 (26.35)	78 (20.10)	
Smoking status, *n* (%)				0.772
Never smoker	246 (44.32)	72 (43.11)	174 (44.85)	
Former smoker	103 (18.56)	34 (20.36)	69 (17.78)	
Current smoker	206 (37.12)	61 (36.53)	145 (37.37)	
Cardiovascular disease, *n* (%)				0.325
Yes	90 (16.22)	31 (18.56)	59 (15.21)	
No	465 (83.78)	136 (81.44)	329 (84.79)	
GPAQ, *n* (%)				0.818
≥600	343 (61.80)	102 (61.08)	241 (62.11)	
<600	212 (38.20)	65 (38.92)	147 (37.89)	
Diabetes, *n* (%)				0.082
Yes	96 (17.30)	36 (21.56)	60 (15.46)	
No	459 (82.70)	131 (78.44)	328 (84.54)	
Hypertension, *n* (%)				0.825
Yes	313 (56.40)	93 (55.69)	220 (56.70)	
No	242 (43.60)	74 (44.31)	168 (43.30)	
Drinking, *n* (%)				0.545
Yes	399 (71.89)	123 (73.65)	276 (71.13)	
No	156 (28.11)	44 (26.35)	112 (28.87)	
Sarcopenia, *n* (%)				0.465
No	484 (87.21)	143 (85.63)	341 (87.89)	
Yes	71 (12.79)	24 (14.37)	47 (12.11)	
GNRI, *n* (%)				0.314
≤98	71 (12.79)	25 (14.97)	46 (11.86)	
>98	484 (87.21)	142 (85.03)	342 (88.14)	

### 3.2 Development of the nomogram prediction model

**FIGURE 2:**
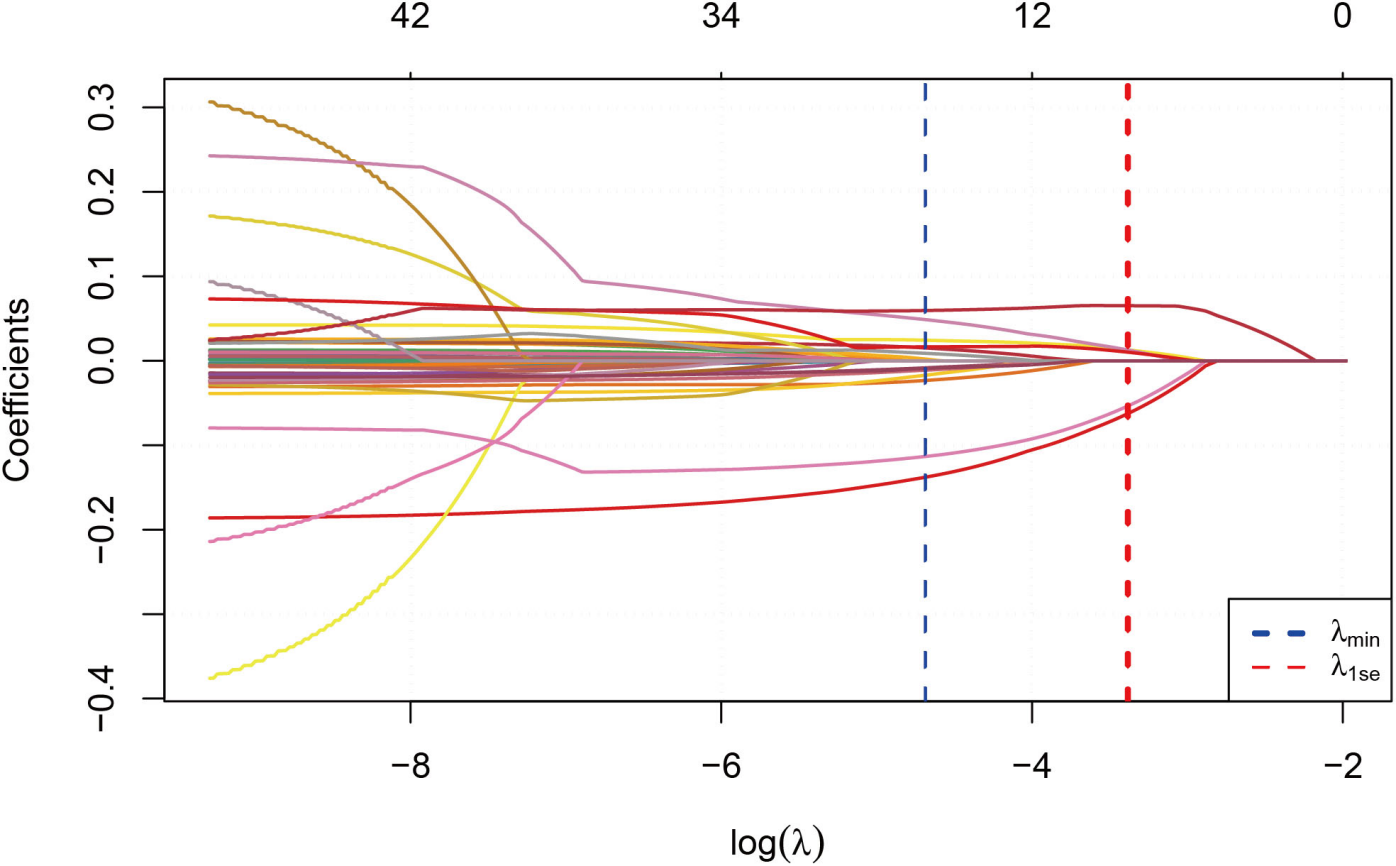
LASSO regression model cross-validation plot.

**FIGURE 3:**
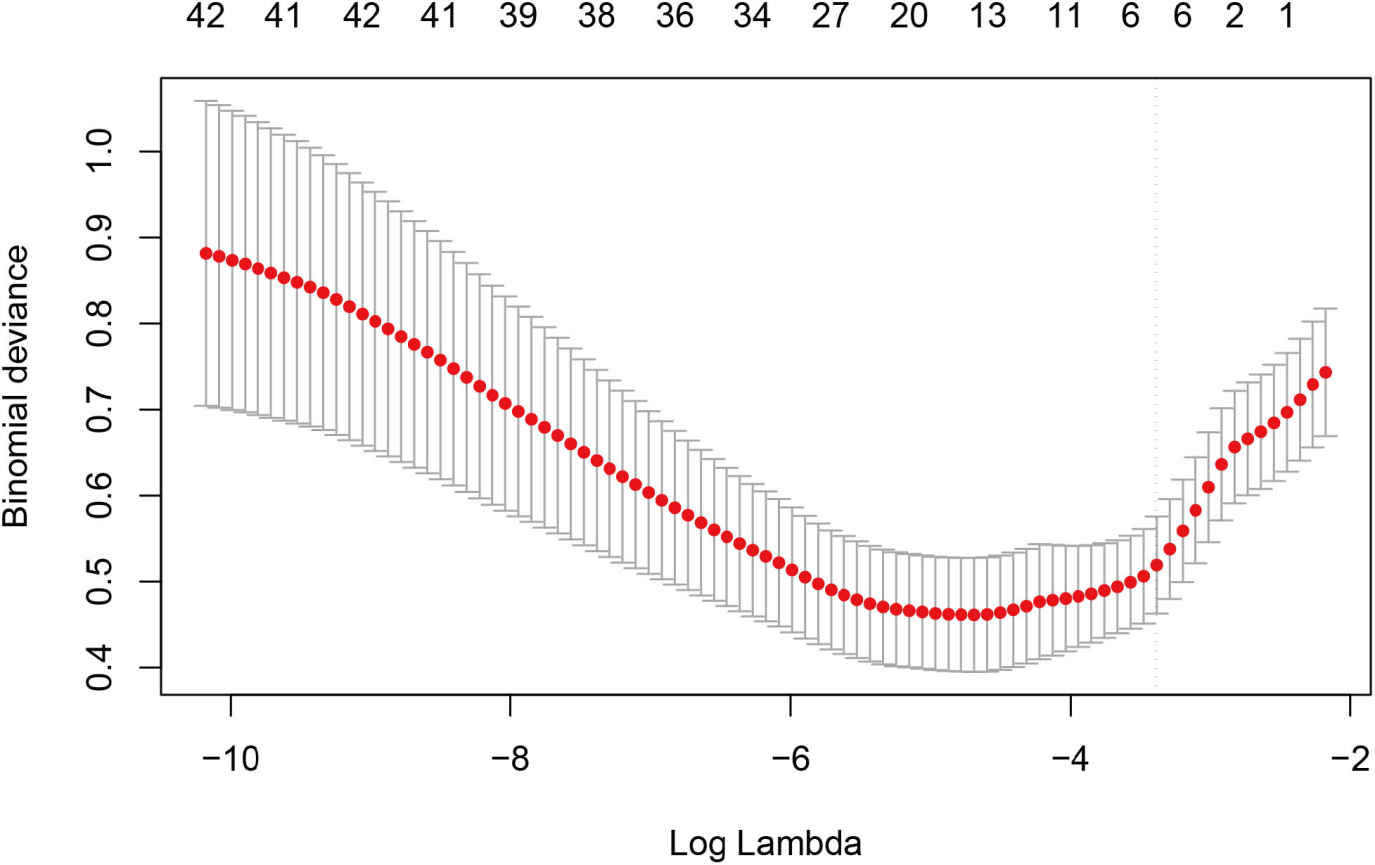
Coeffïcient profïle plot of predictors.

We performed correlation analysis on 42 variables to determine their relationship with sarcopenia in COPD patients. Figure 2 illustrates the coefficient curves for different regularization paths. As the λ value increased, the coefficients gradually approached zero, indicating a reduction in model complexity as regularization strengthened. Figure 3 shows the change in Mean-Squared Error (MSE) at different λ values. As the λ value increased, the MSE exhibited varying trends. LASSO regression analysis was performed, and parameters were optimized using the minimum standard error and 10-fold cross-validation (lambda = 0.0372 at 1 standard error).

Six optimal variables were selected, including gender, HbA1c, height, BMI, MLR, and WWI. After incorporating these variables into multivariate logistic regression analysis, Table 2 shows the following statistically significant variables: Female (OR = 0.003, 95%CI(0.001,0.017), *P* < 0.001), Height (OR = 0.758, 95%CI(0.692,0.830), *P* < 0.001), BMI (OR = 1.092, 95%CI(1.027,1.162), *P* = 0.005), and WWI (OR = 5.830, 95%CI(2.551,13.323), *P* < 0.001).

**TABLE 2:** Multivariate logistic analysis based on training group.

Characteristic	OR (95%CI)	*P-*value
**Gender**		
Male	Ref.	
Female	0.01 (0.01,0.02)	<0.001
Glycohemoglobin (%)	1.24 (0.91,1.68)	0.174
Height (cm)	0.76 (0.70,0.83)	<0.001
BMI (kg/m^2^)	1.09 (1.03,1.16)	0.005
MLR	3.91 (0.30,51.51)	0.300
WWI (cm/√kg)	5.83 (2.55,13.32)	<0.001

**FIGURE 4:**
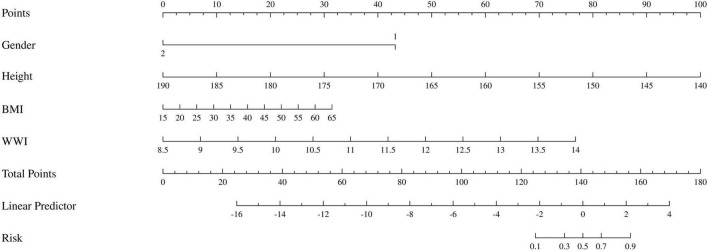
The nomogram of patients with COPD complicated by sarcopenia. Gender: 1 represents male, 2 represents female.

Based on the variables with *P* < 0.05 from the multivariate logistic regression, we developed the nomogram for predicting sarcopenia in COPD patients, as shown in Figure 4. The nomogram model integrated multiple key variables, with each variable represented along a specific scale, and its value associated with a corresponding score. The cumulative score of all variables was used to generate a linear prediction, which was mapped to a “risk” scale (from 0 to 1) to indicate the likelihood of an event occurring. For example, for a male COPD patient with a height of 154 cm, BMI of 28, and WWI of 11.11, the total score calculated by the nomogram was 156 points, with a sarcopenia occurrence rate of approximately 90%. In fact, this patient already had concurrent sarcopenia.

**FIGURE 5:**
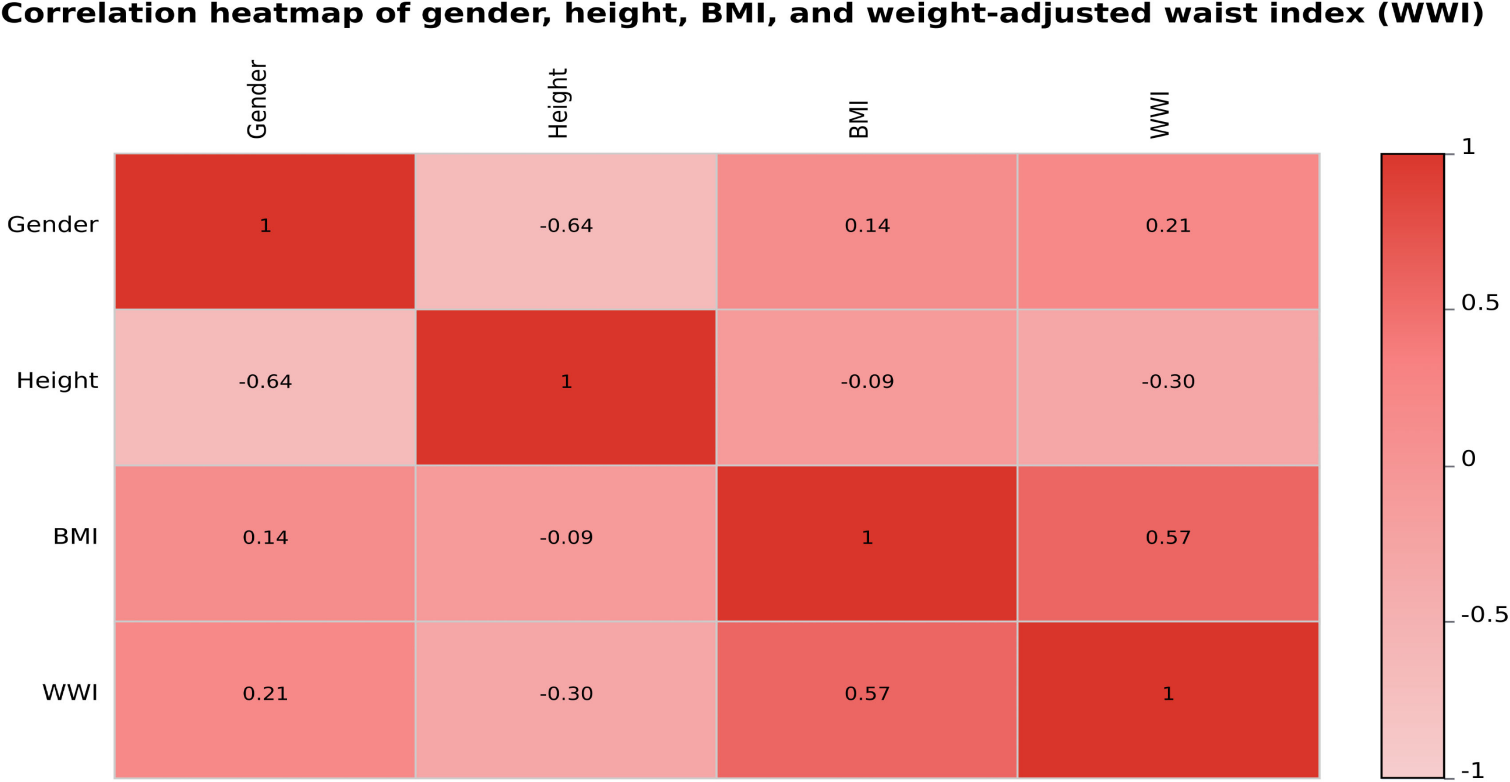
Spearman correlation heatmap among gender, height, BMI, and WWI.

To further assess the potential multicollinearity among the four variables included in the final predictive model (gender, height, BMI, and WWI), a Pearson correlation analysis was conducted, and the results were visualized as a correlation heatmap (Figure 5). As shown in the figure, the pairwise correlation coefficients ranged from –0.64 to 0.57, with the highest correlation observed between BMI and WWI (r = 0.57). The correlations among the remaining variables were weak or negligible, indicating that these four predictors exhibited good statistical independence.

### 3.3 Discriminatory ability and performance of the nomogram

**FIGURE 6:**
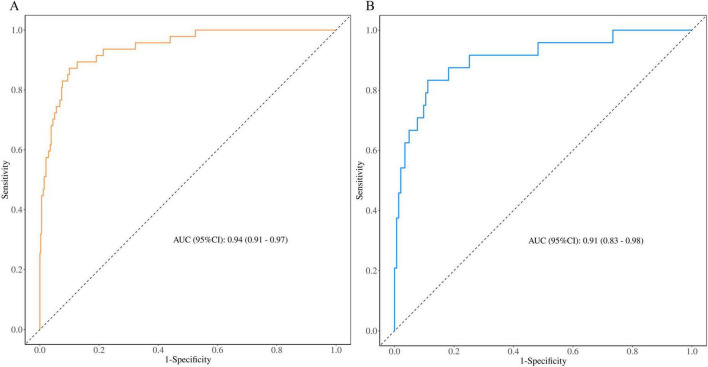
Receiver operating characteristic curves of nomogram based on the data of the training group **(A)** and validation group **(B)**.

To assess the predictive performance of the nomogram model, we plotted ROC curves for both the training and validation groups (Table 3). Figure 6A shows the ROC curve for the training group, demonstrating strong discriminative ability and predictive accuracy, with an AUC of 0.94 (95%CI (0.91,0.97), sensitivity = 0.90, specificity = 0.87, positive predictive value = 0.98, negative predictive value = 0.55). Figure 6B shows the ROC curve for the validation group, with the model also displaying strong discriminatory ability and predictive accuracy, AUC of 0.91 (95%CI (0.83,0.98), sensitivity = 0.94, specificity = 0.67, positive predictive value = 0.94, negative predictive value = 0.67).

**TABLE 3:** Performance of the predictive model in the training and validation groups.

Data	AUC (95%CI)	Accuracy (95%CI)	Sensitivity (95%CI)	Specificity (95%CI)	PPV (95%CI)	NPV (95%CI)	Cut off
Training group	0.94 (0.91,0.97)	0.90 (0.86,0.93)	0.90 (0.87,0.93)	0.87 (0.78,0.97)	0.98 (0.97,1.00)	0.55 (0.43,0.66)	0.197
Validation group	0.91 (0.83,0.98)	0.90 (0.85,0.94)	0.94 (0.91,0.98)	0.67 (0.48,0.86)	0.94 (0.91,0.98)	0.67 (0.48,0.86)	0.197

### 3.4 Validation of the nomogram

**FIGURE 7:**
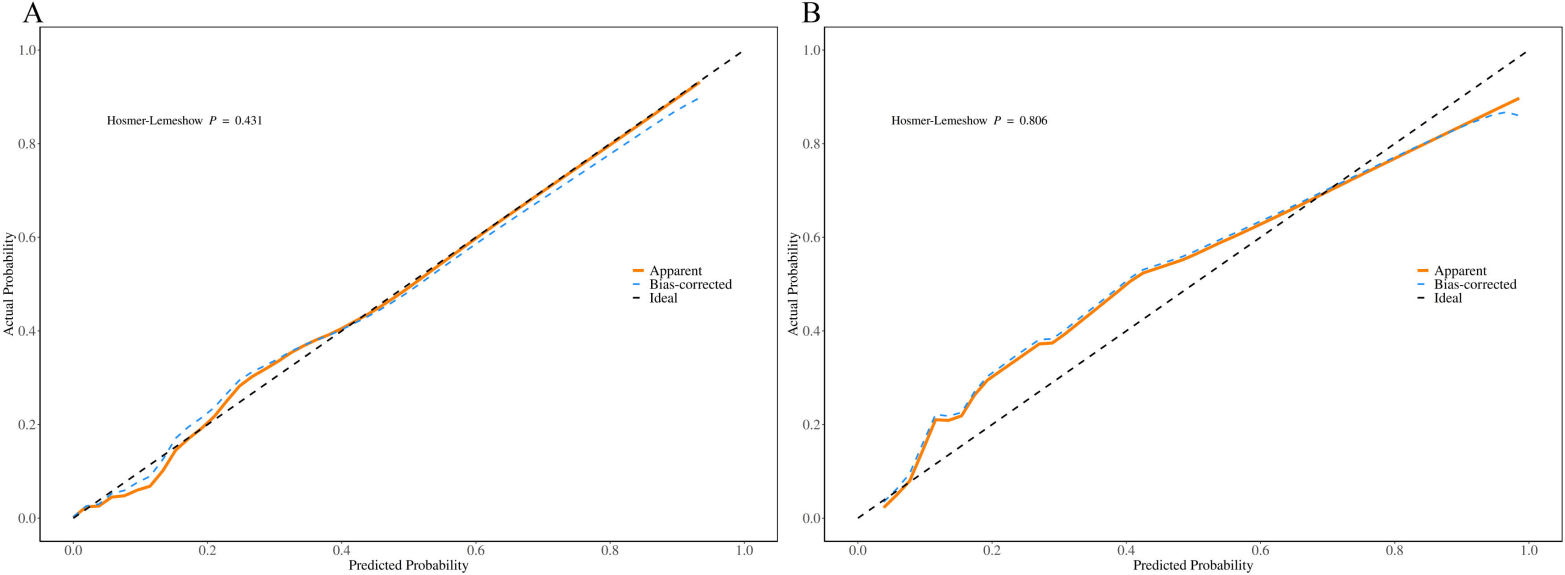
Calibration curve of the nomogram based on the data of training group **(A)** and validation group **(B)**.

We generated calibration curves for the training and validation groups using the 1000 bootstrap resampling technique in order to further confirm the precision of the model’s probability estimation. The calibration curves for the training and validation groups are displayed in Figures 7A, B and they nearly match the diagonal. According to the results of the Hosmer-Lemeshow test, the training and validation groups had *P*-values of 0.431 and 0.806, respectively.

### 3.5 DCA curves

**FIGURE 8:**
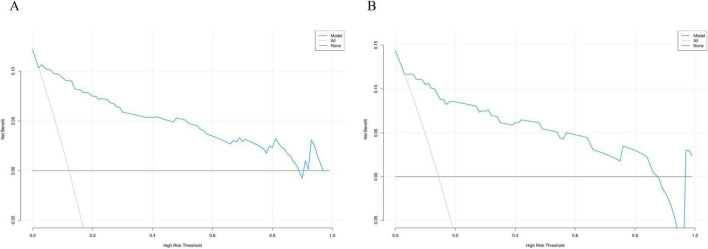
Decision curve analysis curve of the nomogram based on the data of training group **(A)** and validation group **(B)**.

To evaluate the clinical utility of the nomogram model, we plotted the DCA curves. The DCA curves for the training group (Figure 8A) and validation group (Figure 8B) indicate that the model provides a good net benefit when applied with intervention measures within a probability range of 0.05 to 0.8.

## 4 Discussion

This study developed a nomogram prediction model that uses simple demographic and biochemical factors to predict the risk of sarcopenia in COPD patients. Through regression analysis with LASSO and Analysis of logistic regression using multiple variables, the study identified four key predictive factors: gender, height, BMI, and WWI. The model demonstrated high predictive ability, with AUC values of 0.94 and 0.91 for the training and validation sets, respectively. Internal validation, along with the construction of calibration curves and DCA curves, further supported the model’s reliability and clinical applicability.

According to the NIH (24) and the European Consensus on Sarcopenia Diagnosis (25), the diagnosis of sarcopenia, DXA is regarded as the gold standard. However, the European diagnostic guidelines point out several limitations of DXA: First, while DXA is a non-invasive testing method, its high cost limits its widespread use in community settings. Second, DXA may struggle to accurately distinguish between muscle and fat tissue in individuals with low muscle mass or edema, leading to reduced measurement accuracy. Finally, DXA involves low-dose radiation, and its prolonged or frequent use may pose potential health risks, particularly for older adults or individuals who require frequent monitoring. Therefore, the development of a simple and practical early prediction tool is of paramount importance.

Currently, various screening tools have been developed for sarcopenia in COPD patients, but their applicability and effectiveness remain challenged. A study on the risk factors for sarcopenia in COPD patients proposed a risk prediction model, expressed as: lgitP = −4.323 + 1.094 × X_*age*_ + 0.914 × X_*BMI*_ + 0.837 × X _*smoking history*_ + 0.846 × X_*annual acute exacerbation frequency*_ + 0.410 × X _*CAT score*_−0.069 × X_*FEV*1/FVC%_ + 0.561 × X_*COPD duration*_. However, the model is overly formulaic and complex, with an AUC of 0.756 and specificity of 0.632, indicating low predictive value (26).

In addition, another study investigated whether ultrasonographic evaluation of the rectus femoris muscle in COPD patients could predict sarcopenia and developed a corresponding nomogram model. The model indicated that rectus femoris thickness, cross-sectional area, age, and BMI could predict the occurrence of sarcopenia in COPD patients. However, this study did not consider important factors such as biochemical markers, nutritional status, and daily physical activity (27).

In our study, four key predictive factors were identified: gender, height, BMI, and WWI. From the gender perspective, as age increases, men typically experience a physiological decline in testosterone levels (28). In COPD patients, hypoxemia (29), obesity (30), and glucocorticoid therapy can lead to hypogonadism, resulting in reduced testosterone levels (31). Studies have shown that testosterone treatment increases both low and high concentrations of type I muscle fibers, as well as high concentrations of type II muscle fibers (32). Additionally, testosterone has a high rate of amino acid reutilization within cells, promoting protein synthesis and increasing muscle fiber size (33). In men with hypogonadism undergoing testosterone treatment, significant increases in muscle mass have been observed, with skeletal muscle hypertrophy driven by increased muscle protein synthesis rates (34). In a 3-year double-blind trial, older adult men receiving testosterone replacement therapy showed greater improvements compared to the placebo group (34). Although women experience muscle loss after menopause due to reduced estrogen levels, the impact is generally less pronounced than the effect of testosterone on sarcopenia in men (35). Furthermore, studies have suggested that as COPD progresses, male patients may experience a greater reduction in physical activity, which is a significant risk factor for sarcopenia (36). This finding is consistent with our results.

Our nomogram results indicate that the risk of sarcopenia in male COPD patients is significantly higher than that in females. Based on this finding, we recommend that clinical practice place more emphasis on male COPD patients and implement a range of effective interventions. In the early stages of the disease, it is crucial to regularly assess muscle mass and physical function in male patients. Therefore, male COPD patients should undergo regular physical assessments and receive personalized intervention strategies under the guidance of their healthcare providers. Early pharmacological treatments and non-pharmacological interventions can effectively delay further muscle loss and reduce the risk of developing sarcopenia (16).

Our study also found that as height decreases, the risk of sarcopenia in COPD patients increases. Aging generally leads to a gradual decrease in height (37), particularly around the age of 45, when a noticeable decline begins at a rate of approximately 0.09% per year (38). Significant height loss may indicate underlying skeletal health issues, such as osteoporosis (39) or vertebral fractures (40). Therefore, older adult individuals are at increased risk of height reduction and should be particularly mindful of their bone health. Ji et al. found that individuals with greater height loss are more likely to be diagnosed with sarcopenia (41). A nomogram prediction model for sarcopenia in diabetes was developed in a study with 1,246 diabetic patients, and the results showed that a decrease in height is associated with a higher risk of sarcopenia in diabetic patients (42). In a large sex-stratified study conducted in Korea, stature was identified as an independent clinical risk factor for sarcopenia in both men and women (43). Furthermore, another investigation demonstrated that a loss of more than 4 cm in height is associated with a markedly increased risk of developing sarcopenia (OR = 2.676; 95% CI = 1.122–6.284) (44). Our nomogram model also indicates that height is an important risk factor in the current study, which is consistent with previous research.

In addition, BMI is closely related to the occurrence of sarcopenia in COPD patients. A higher BMI often signifies obesity. Research has indicated that with increasing age, body fat content rises, resting metabolic rate decreases, and inflammation intensifies, all of which promote fat accumulation, increased waist circumference, and ultimately lead to sarcopenia, a phenomenon referred to as “sarcopenic obesity” (45). Moreover, obesity may further exacerbate the development of sarcopenia (46). Similarly, our study demonstrated a consistent increase in sarcopenia scores with rising body mass index (BMI), indicating a positive correlation between higher BMI and an elevated risk of developing sarcopenia.

Our nomogram also includes the WWI as a factor. Previous studies have clearly indicated a significant negative correlation between WWI and ASM (*r* = −0.511, *P* < 0.001) (47). In a Korean study involving 5,983 individuals, a high WWI was associated with high fat mass, low muscle mass, and low bone mass, suggesting that WWI could serve as a comprehensive indicator of body composition (48). In another study examining the correlation between WWI and sarcopenia, it was found that after adjusting for confounders, for each increase of 1 unit in WWI, the risk of sarcopenia in men increased by 14.55 times (49). Moreover, in individuals with diabetes, a higher weight-adjusted waist index (WWI) has been identified as an independent risk factor for sarcopenia (OR = 1.836; 95% CI: 1.216–2.772) (50). Consistently, our multivariate logistic regression analysis revealed that for each unit increase in WWI, the risk of developing sarcopenia increased by 4.83 times.

Based on the nomogram model we developed, we recommend early identification and intervention for COPD patients in clinical practice to reduce the risk of sarcopenia (51). First, regular aerobic exercise and resistance training have been shown to play a significant role in maintaining and enhancing muscle strength (52). It is recommended that patients engage in appropriate activities such as walking, cycling, or light resistance training, which not only help improve muscle mass but also enhance endurance and daily functional capacity. Second, nutritional support is another key factor in preventing sarcopenia. Studies have demonstrated that vitamin D plays a crucial role in maintaining muscle function, with vitamin D deficiency being linked to increased muscle weakness and sarcopenia (53). Therefore, ensuring adequate vitamin D levels through supplements or sunlight exposure is essential. Lastly, weight management is particularly important for male COPD patients (54). Maintaining a healthy weight not only reduces the risk of sarcopenia but also improves overall health. Through a balanced diet and moderate exercise, patients can avoid excessive weight loss or obesity. Additionally, avoiding prolonged sitting and bed rest is an effective strategy to prevent muscle wasting (55).

The nomogram prediction model for sarcopenia in COPD patients developed in this study assists clinicians in quickly identifying high-risk COPD patients with sarcopenia under non-invasive and low-cost conditions. The model performs exceptionally well, with an AUC of 0.94 for the training set and 0.91 for the validation set, providing strong evidence for early intervention. The main advantages of this model include: (1) all the indicators used are based on physical measurements, which do not require complex equipment; (2) compared to existing methods that heavily rely on imaging examinations, this model clearly identifies the relevant risks of sarcopenia in COPD patients through simple physical measurements; (3) the nomogram provides an intuitive, convenient, and personalized risk prediction tool with strong potential for wide application.

The nomogram developed in this study demonstrates good clinical usability and potential for broader implementation. Based on four simple, non-invasive indicators (gender, height, BMI, and WWI), it requires no laboratory or imaging tests and is suitable for use in primary care, rehabilitation, and community health settings. Its intuitive interface allows healthcare providers to rapidly assess sarcopenia risk in COPD patients, enabling early detection and stratification. This supports the development of individualized nutritional, physical, and therapeutic interventions and holds significant value in chronic disease management. Despite the high predictive accuracy of the proposed nomogram, several limitations should be acknowledged. First, the retrospective design may introduce selection bias; therefore, large, multi-centre prospective studies are warranted to verify the model’s generalizability and stability. Second, all data were obtained from a single source (NHANES), underscoring the need for external validation using independent, multi-centre cohorts. Third, although age, smoking status, serum albumin level and diabetes history were included in the initial variable pool, they were excluded during LASSO selection because of partial information overlap with other predictors or limited incremental predictive value in the training set. This statistical exclusion does not negate their biological relevance, and their prognostic contribution should be clarified in future prospective or multi-centre investigations. Finally, this study did not differentiate COPD phenotypes, which limits the ability to assess subtype-specific risk patterns. Moreover, data on participants’ use of systemic corticosteroids, inhaled corticosteroids, and GOLD classification were incomplete or unavailable, further restricting the granularity of COPD-related risk stratification. Future studies should address these factors to refine the model’s applicability and predictive accuracy.

## 5 Conclusion

An effective and practical technique for the early detection of sarcopenia in COPD patients was created by this study using a nomogram model based on gender, height, BMI, and WWI. The model demonstrates excellent predictive ability and clinical utility, offering valuable guidance for early intervention and improving patient prognosis.

## Data Availability

The raw data supporting the conclusions of this article will be made available by the authors, without undue reservation.
